# Systemic Sclerosis Association with Malignancy

**DOI:** 10.1007/s12016-022-08930-4

**Published:** 2022-09-19

**Authors:** Gemma Lepri, Martina Catalano, Silvia Bellando-Randone, Serena Pillozzi, Elisa Giommoni, Roberta Giorgione, Cristina Botteri, Marco Matucci-Cerinic, Lorenzo Antonuzzo, Serena Guiducci

**Affiliations:** 1 Department of Experimental and Clinical Medicine, University of Florence, and Division of Rheumatology, AOUC & Scleroderma Unit, Florence, Italy; 2 Medical Oncology Unit, Careggi University Hospital, Florence, Italy; 3 Unit of Immunology, Rheumatology, Allergy and Rare Diseases (UnIRAR), IRCCS San Raffaele Hospital, Milan, Italy; 4 Medical Oncology, Department of Experimental and Clinical Medicine, University of Florence, Florence, Italy

**Keywords:** Systemic sclerosis, Malignancy, Cancer, Risk factors, Autoantibodies

## Abstract

The association of systemic sclerosis (SSc) and cancer is well known from several decades suggesting common genetic and environmental risk factors involved in the development of both diseases. Immunosuppressive drugs widely used in SSc may increase the risk of cancer occurrence and different SSc clinical and serological features identify patients at major risk to develop malignancy. In this context, among serological features, presence of anti-RNA polymerase III and anti-topoisomerase I autoantibodies seems to increase cancer frequency in SSc patients (particularly lung and breast cancers). Lung fibrosis and a long standing SSc pulmonary involvement have been largely proposed as lung cancer risk factors, and the exposure to cyclophosphamide and an upper gastrointestinal involvement have been traditionally linked to bladder and oesophagus cancers, respectively. Furthermore, immune checkpoint inhibitors used for cancer therapy can induce immune-related adverse events, which are more frequent and severe in patients with pre-existing autoimmune diseases such as SSc. The strong association between SSc and cancer occurrence steers clinicians to carefully survey SSc patients performing periodical malignancy screening. In the present review, the most relevant bilateral relationships between SSc and cancer will be addressed.

## Introduction

Systemic sclerosis (SSc) is characterized by small vessel vasculopathy, immune dysregulation with production of specific autoantibodies, and fibrosis of skin and internal organs [1]. The disease significantly impacts on patients’ quality of life and on life expectancy with a standardized mortality ratio estimated between 1.5 and 7.2 [2–5]. Today, interstitial lung disease (ILD) and pulmonary arterial hypertension (PAH) are the most frequent causes of death [6–8]; however, infections, cancers, and cardiovascular diseases are the most frequent non SSc-related causes of death [4, 7]. In the EUSTAR (European Scleroderma Trial and Research Group) database, in more than half of cases, ILD and PAH were responsible for a third of all causes of death [4] followed by myocardial involvement (14%) and renal crisis (4%). In 41% of all cases, death was due to infections and cancers. Among all neoplasms, non-small cell lung cancer was the most frequent (11/30) followed by breast (4/30), small cell lung (2/30), and colon-rectal and hepatocellular cancer (both 2/30). Authors reported that other solid (renal cell carcinoma, pancreas carcinoma, angiosarcoma, neuroendocrine cancer, and oesophageal cancer) and haematological cancers (acute myeloid leukaemia, lymphoma, multiple myeloma) were rarer in the SSc [4]. More recently, the analysis of 3700 deaths from two detailed databases confirmed SSc cardiopulmonary involvement as the major cause of death [7]. Furthermore, besides the high mortality rate associated with cardiac disease and respiratory failure due to ILD, SSc patients had a fivefold higher rate of infectious deaths compared to the general population. In addition, authors confirmed cancers as a frequent cause of mortality among SSc patients (about 10% of all death certificates). Also in this study, lung cancer was the most frequent, but authors did not report an increased risk of death from cancer than the general population as also reported in other studies [7, 9–11].

Altogether these data suggest that cancers may be a cause of death in SSc patients. In addition, the risk for cancer seems to be higher within the first 12 months of the initial SSc diagnosis suggesting that SSc might represent a paraneoplastic event in some patients. Previous studies suggested an association between the sites affected by cancer and the presence of fibrosis, particularly regarding lung and skin [9, 10]. Furthermore, the presence of a strong association between SSc and cancers may suggest that common genetic and environmental risk factors may be involved in the development of both diseases [12]. Some SSc pathophysiological mechanisms, such as immune and vascular dysregulation, exuberant fibrogenesis, and oxidative stress, are involved in cancer development, although the link of SSc and oncogenesis remains unknown [13]. In addition, as previously mentioned, cancers in SSc seem to be more frequent in sites affected by an exuberant fibrosis, suggesting that a persistent inflammation leading to fibrosis may represent an underlying mechanism of carcinogenesis [12, 14]. However, also immune dysregulation, acquired genetic damage, and prolonged immunosuppressive treatments are suspected as predisposing mechanism for cancer development in SSc [12, 15, 16]. Recently a pathogenetic role of abnormal B cell function has also been suggested in SSc and this datum could explain the increased lymphoma risk in SSc patients [17, 18]. In addition, a biphasic association between SSc and neoplasia has been recently confirmed [13] suggesting distinct pathogenetic mechanisms. When cancer occurs within the first 5 years of SSc onset, probably it may be considered the first pathological event that could trigger an immune response leading to rheumatic autoimmune disease. In the other case, the trigger is probably linked to SSc disease: chronic inflammation, use of immunosuppressants, and genetic abnormalities with mesenchymal dysfunction may be responsible for occurrence of cancers [13, 19].

It should also be pointed out that SSc may arise as a consequence of anti-cancer therapies. The development of immune checkpoint inhibitors (ICIs) targeting cytotoxic T lymphocyte antigen 4 (CTLA-4) and programmed cell death-1/ligand (PD-1/PD-L1) significantly improved treatment of some cancers allowing a longer remission in those cases that previously were considered untreatable [20]. However, ICIs are associated with the development of immune-related adverse effects (irAEs) and/or disease exacerbations in patients with pre-existing autoimmune disease (PAD). The knowledge of adverse inflammatory effects of ICIs has allowed a significant improvement in the understanding of autoimmune diseases pathogenesis as well as the development of therapeutic strategies involving immune receptor inhibitors for their treatment.

## Epidemiological Data on More Frequent Cancers in SSc Patients and Risk Factors

The association between SSc and cancer is well known. As shown by different studies, the incidence of cancers in SSc patients ranges from 3.6 to 10.7% according to different populations with a standardized incidence ratio (SIR) between 0.75 and 2.73. Studies and meta-analysis reported a higher incidence in men than in women and lung cancer as the most common malignancy among SSc patients [9–11, 21–26]. Data from a recent analysis of 1.727 SSc patients reported a cancer incidence of 1.3% and a prevalence of 14.2% highlighting the economic burden of malignancy in SSc. Authors confirmed a higher risk for cancer in SSc subjects compared to general population matched for sex and age, particularly for lung, breast, and cutaneous melanoma cancers. In addition, this study showed an increased SIR for early cancer occurrence in the course of the rheumatic disease, particularly within the first 5 years from SSc diagnosis [19]. Cancers seem to occur independently from traditional cancer risk factors as smoking and immunosuppressive drugs; furthermore, patients with cancer presented a higher mortality rate. However, the association of malignancy in SSc and the traditional cancer risk factors still remains controversial, as other studies reported a strong correlation of malignancy occurrence and heavier smoking, similar to that observed in general population [27, 28]. An increased incidence of malignancies has been reported in SSc patients enrolled in the nationwide Danish National Registry: 222/2040 cases of cancer were diagnosed after SSc diagnosis (median follow-up of 6.4 years) [12]. The SIRs were 1.5 (95% CI 1.3–1.7) for all cancers with a significant higher risk in men than in women. This evidence confirmed that SSc is a risk factor for the development of smoking- and alcohol-related neoplasms, particularly lung cancer. Among all haematological malignancies, the risk was higher for non-Hodgkin lymphoma and leukaemia and authors also reported a high incidence of immune-related cancers (SIR = 1.4, 95% CI 1.0–1.9) [12]. Data from another cohort study in southwest England confirmed an increased overall malignancy risk [3.15 (95% C.I. 1.77–5.20)] reporting cancers in 15/68 SSc patients: in most cases (86.7%), the diagnosis of cancer followed the SSc onset. In this population, a significant increased risk to develop haematological malignancies was observed [29]. A meta-analysis evaluated cancer risk in SSc patients, reporting a pooled SIRs for overall risk of malignancy of 1.41 (95% CI 1.18–1.68), higher in men than in women [1.85 (95% CI 1.49–2.31) vs 1.33 (95% CI 1.18–1.49) respectively]. Data from this meta-analysis reported an increased risk in the occurrence of lung, liver, bladder, and hematologic cancers [30]. Out of 340 enrolled patients, 19 cancers were found in 15 patients: bladder cancer was the most common and associated to cyclophosphamide exposure, followed by breast cancer [28].

As mentioned above, many studies showed lung cancer as the most frequent among SSc patients with longstanding pulmonary involvement, thus hypothesizing that ILD might be a condition predisposing to the occurrence of cancer [10, 11]. Recently, data from a single-centre study in China compared 19 SSc patients with diagnosis of lung cancer with 79 SSc control subjects. In this population, the presence of ILD was confirmed as a risk factor for lung cancer development together with a family history of neoplasm [31]. Recently, the demographic and clinical characteristics of 12 SSc patients with lung cancer were analysed: all patients were female and only 8 presented ILD, suggesting that lung fibrosis does not represent neither the unique risk factor for cancer nor a necessary precondition for cancer occurrence. None of all enrolled patients had history of smoking [32]. From 1999 and 2001, the incidence of lung cancer was studied in 318 SSc Caucasian subjects and it was found to be quite higher (about 5%) than in previous studies. Adenocarcinoma was the most common lung cancer detected in this study [33] and authors confirmed a significant increased incidence of lung cancer in SSc population when compared with general population of the same geographic area and matched for sex and gender. 16.1% of SSc male patients presented lung cancer (vs 6.2% in the general population) and 3.8% of SSc females (vs 0.7%). The study reported lung cancer as more frequent in patients with a longer disease duration and with a lower age at SSc diagnosis. Anti-topoisomerase I antibodies (anti-topo I abs) and a SSc lung involvement (particularly the reduction of forced vital capacity) were shown to be associated with cancer occurrence, also by the logistic regression analysis [33]. In a single-centre observational study on 210 SSc patients (81 with a diffuse cutaneous SSc, dcSSc), 10% of patients developed cancer during a follow-up of about 6 years [34]. Out of all 21 diagnosed cancers, the most common were represented by lung and breast cancers and an association between the occurrence of neoplasm and a story of renal crisis and the positivity of anti-topo I abs was reported, as already suggested [33, 34]. Lung cancer was the most common malignancy also in the analysis of 2053 Taiwanese SSc patients. In this cohort, the incidence of cancer was reported to be 6.9/1000 person-year and lung cancer was confirmed to be the most frequent with a SIR of 4.20. In female SSc patients, lung cancer was common as the breast one [35]. According to different data, the incidence of lung cancers in SSc patients may range from 0 to 4.2% [11, 29, 36, 37] probably reflecting the different scleroderma clinical subsets of enrolled patients with a consequent different susceptibility to develop cancer [33]. However, a significant increased risk of lung cancer in SSc population compared to general subjects has been recently confirmed by a further meta-analysis on 12.218 patients revealing its higher frequency particularly in male subjects [38].

The correlation between SSc and breast cancer is variable and debated with discordant results probably due to study methods and/or population heterogeneity [12, 22, 39, 40]. Among studies demonstrating an association between breast neoplasm and SSc, already in 2004, a 3.9% cases of breast cancer (8 women) out of 203 SSc patients enrolled between 1990 and 2002 were reported [41]. In some case reports, authors confirmed a close temporal association between SSc and breast cancer [42–45]. These results suggest a pathophysiological link between the two pathologies and a paraneoplastic nature of SSc. However, this hypothesis remains still to be confirmed as authors did not report a SSc amelioration after cancer treatment or surgical removal [41]. The link between SSc and breast cancer was also reported by Colaci et al. showing a significant higher incidence (12 patients, 11 women and 1 man) of breast cancer among 318 SSc patients than in sex–age-matched general population from the same geographic area with a SIR of 2.1 (95% IC: 1.13–3.90; *p* < 0.01) [39]. This study did not find differences in clinical and serological features between SSc patients with breast cancer and those without, except for a relatively shorter disease duration at the time of breast cancer diagnosis in the first group [39]. A more recent observational retrospective multicentre study enrolled 33 SSc women with breast cancer from January 2017 to December 2019, without confirming the temporal relationship between the two disorders [46]. In fact, 54.5% of these patients presented breast cancer before SSc onset (with a median of 5 years between the two diseases onset) and in 45.5% subjects SSc was diagnosed before the cancer with a median of 8 years. In this population, 75% of invasive cancers were positive for hormone receptors, about 28% had HER2 positivity, and 19% were triple negative. Regarding SSc features, more than 50% of SSc patients had ILD and all 6 cases of death were SSc-related due to PAH [46]. At SSc onset, an association between breast cancer, age, and anti-RNA polymerase III (anti-RNA Pol III) antibodies was detected by a multivariable logistic regression (OR 1.07, *p* < 0.001 and OR 4.28, *p* = 0.018 respectively) [19]. To note that calcium channel blockers, widely used in SSc patients, have been suspected to be a risk factor in the development of breast cancer both for ductal and lobular cancers [19, 47, 48]. However, the real relationship between these drugs and breast cancer remains uncertain and other studies provided strong evidence of no association [49, 50]. A role of hormones has been suggested both in SSc and in breast cancer, in particular the increased level of prolactin and decreased levels of dehydroepiandrosterone sulfate [51, 52].

In SSc, an association with oesophageal and bladder cancer has been described. The first seems to correlate with the typical SSc upper gastrointestinal involvement characterized by dysmotility and gastro-oesophageal reflux that may lead to Barret’s oesophagus increasing the risk of oesophagus dysplasia [53]. In fact, data from the analysis of the EUSTAR database revealed that out of 46 SSc patients with Barret’s oesophagus, 4 presented a high-grade dysplasia after a follow-up of 3 years and among those, in one patient a cardial oesophageal adenocarcinoma was diagnosed [54]. Regarding bladder cancer, many studies reported an increased risk of its occurrence with the use of cyclophosphamide [28, 55, 56]. However, recently Lertphanichkul and Smith [57] reported 11 cases of genitourinary cancers among 125 SSc patients and 6/11 were bladder cancers. However, none of these 6 patients has been treated with cyclophosphamide, suggesting that this alkylating agent is not the unique risk factor for bladder cancer in SSc subjects.

In SSc, an increased risk of non-solid cancers has been described and an association with haematological cancers has been reported [26, 30, 55]. One hundred-thirty cases of haematological neoplasms in SSc were evaluated from 1954 to 2017 [58]. In most cases, patients presented haematological cancer occurrence close to SSc diagnosis, in 30% the neoplasm was diagnosed within 5 years of SSc onset and in others 30% of patients the temporal relationship was so close to suspect a paraneoplastic nature of SSc [58, 60]. The most common cancer was lymphoma (B cells non-Hodgkin lymphoma), followed by leukaemia, multiple myeloma, and myeloproliferative disorders [58]. However, the increased risk of lymphoma among SSc patients is still a matter of debate. A study on more than 200 Hungarian SSc patients reported non-Hodgkin lymphoma as a rare event in SSc, although with an incidence about two times higher than in general population [17]. Also this study showed an early occurrence of lymphoma in the course of SSc, occurring within 2 years from the onset of the rheumatic disease [17, 59]. Evaluating 251 Italian SSc patients, Vettori et al. described a prevalence of 0.49% of non-Hodgkin lymphoma. In addition, authors conducted a literature review reporting a certain correlation between non-Hodgkin’s lymphoma and old age, female sex and dcSSc [60].

According to data from a previous study of Derk et al. [61] an increased frequency of tongue cancer in SSc patients has also been reported. Among 769 SSc patients, authors showed the presence of oral or pharyngeal carcinoma in 9 (11%) subjects (6/9 tongue cancer). All patients with tongue cancer presented a SSc diffuse subset; the exposure to alcohol and tobacco was reported in 16% of patients and 33% of patients had a family history of cancer [61].

Regarding cutaneous cancer, Morrisroe et al. [19] reported an increased risk of early melanoma [SIR 3.40 (95% CI 1.10–7.93)] without association with traditional risk factors as smoking and immunosuppression [19]. In the study of Olesen et al. [12], an increased frequency of melanoma and cervical cancer was reported. If considered patients after 12 months of follow-up, authors reported an increased number of non-melanoma skin cancer in men (SIRs of 2.4, 95% CI 1.2–4.4) and a previous study already described 5 cases of non-melanoma skin cancers among 69 tumours in 917 enrolled SSc patients. Data from this study showed a SIR of 1.8 (95% CI, 0.02–10.1) in men and of 6.3 (95% CI, 1.7–16.0) in women [9].

In conclusion, reported data showed an increase cancer incidence in SSc population, particularly regarding lung and breast cancer. Among haematological cancers, lymphoma seems to be the more frequent. The cancer occurrence in SSc seems to be characterized by a biphasic trend, developing many years after the diagnosis of SSc or occurring early in the course of the rheumatic disease, often within the first 5 years from its onset. Some authors suggested a certain association between lung fibrosis and lung cancer; however, this datum has not been confirmed by all studies and the pathophysiology link between the two disorders has still to be cleared. The more frequent cancers in SSc patients and their suggested risk factors are reported in Table 1. Data about the association between cancer and SSc-specific autoantibodies will be discussed in the next paragraph.

**Table 1:** More frequent cancers in SSc patients and suggested risk factors

Lung cancer • Long standing pulmonary involvement (SSc interstitial lung disease) • Traditional risk factors as smoking • Longer SSc duration • Lower age at SSc diagnosis • Anti-topoisomerase I antibodies • History of scleroderma renal crisis • Male sex
Breast cancer • Anti-topoisomerase I antibodies, antiRNA polymerase III antibodies • History of scleroderma renal crisis • Temporal association between SSc and breast cancer diagnosis with a shorter SSc duration at time of breast cancer diagnosis • Age at SSc onset
Bladder cancer • Cyclophosphamide exposure
Oesophagus cancer • Upper gastrointestinal involvement with dysmotility and gastroesophageal reflux
Haematological neoplasm • Close relationship with SSc diagnosis • Old age, female sex and diffuse cutaneous subset (for non-Hodgkin lymphoma)
Tongue, oral of pharyngeal cancer • Diffuse subset (tongue) • Traditional risk factors (as alcohol exposure and family history) (tongue)

### SSc-Specific Autoantibodies and Cancer Risk

Among all SSc clinical and serological features, some SSc-specific autoantibodies have been demonstrated to increase the risk for cancer development suggesting a molecular link between autoimmunity and neoplasm. To identify risk factors is essential both to stratify patients into more clinically relevant subsets and to follow-up SSc patients with personalized cancer screening recommendations [62].

In this context, since several years a certain association between anti-RNA Pol III antibodies and an increased risk cancer with a close temporal gap between SSc and neoplasm have been reported [19, 57, 63–65]. In 2014, Moinzadeh et al. [66] reported 154 cancers (7.1%) among 2.177 enrolled patients and confirmed a significant association between anti-RNA Pol III antibodies and a close cancer occurrence (36 months within SSc onset). The incidence of neoplasms was higher in anti-RNA Pol III patients than in those with anti-topo I or anticentromere antibodies (ACA) positivity (14.2% vs 6.3% and vs 6.8% respectively). In this study, breast cancer was reported as more frequent in patients with anti-RNA Pol III antibodies and these antibodies were reported as the only ones that significantly increased the risk of cancer. Comparing to SSc patients negative for anti-RNA Pol III antibodies, those positive were reported to present nearly six times increased risk of developing cancer within 3 years and 19 times more likely to present breast cancer within 36 months of onset of SSc when compared to patients with ACA [66]. Out of 2.383 SSc enrolled patients, Igusa et al. [62] reported neoplasm in 8.6% of patients. Authors did not demonstrate that a diffuse disease subset associated with anti-RNA Pol III antibodies significantly increased the risk of cancer development. Evaluating the risk of neoplasm within the first 3 years of SSc onset, patients with dcSSc were at major risk to develop cancer compared to the general population, and this risk further increased if they presented anti-RNA Pol III abs positivity. Therefore, data from this study also suggested that the risk of malignancy may differ among anti-RNA Pol III patients according to their cutaneous subset: dcSSc anti-RNA Pol III patients were reported to have a higher risk of breast cancer and lcSSc anti-RNA Pol III patients an increased risk of lung cancer. However, this last result was uncertain, given the small numbers of patients with lung malignancy in the enrolled population. Authors also showed a decreased cancer risk in patients with ACA positivity and an increased risk, particularly for breast cancer and melanoma, in patients with limited subset (lcSSc) and “triple negative”, that is lacking ACA, anti-topo-I, and anti-RNA Pol III [62]. Similar findings were already reported by a previous study on 23 SSc patients with cancer demonstrating a difference in SSc median duration at neoplasm onset between patients positive for anti-RNA Pol I/III and those with anti-topo I or ACA positivity. In the first group, a close temporal relationship between cancer and SSc was shown reporting SSc onset within 2 years of malignancy diagnosis. A similar close temporal association with cancer and “triple negative” SSc patients was also shown [63]. In this study, authors investigated the expression of RNA polymerase I and III in cancer tissues reporting an increased nucleolar expression of RNA polymerase III in tumor cells from patients with anti-RNA Pol III antibodies compared to those negative for these antibodies. According to this finding, an association between tumor antigen expression and SSc-specific autoantibody responses may be suggested, advancing the hypothesis of a close relationship between cancer, immune response, and SSc occurrence. Although requiring further validations, according to these data, anti-RNA Pol III antibodies could be considered a marker of malignancy [63]. In 2015, a study reported 168/1.044 (16.1%) SSc patients with cancer and confirmed the higher frequency of cancers in anti-RNA Pol III patients (20.9%) compared to those with ACA (16%), anti-topo I (13.6%), and to SSc subjects negative for these three antibodies (14.9%) [67]. Comparing the two populations, SSc patients with cancers vs SSc subjects without, a significant higher frequency of white race, older age, and anti-RNA Pol III positivity were observed in the first group. The older age at SSc onset was a risk factor both for cancer development and for its occurrence in close temporal relationship with SSc beginning and this last datum was particularly confirmed in patients with anti-topo I positivity and in those negative for anti-RNAP III, anti-topo I, and ACAs. In addition, in this study the close temporal association between cancer occurrence and SSc has been again reported in anti-RNA Pol III patients [67]. A case–control study that collects data from 13 EUSTAR centres confirmed a higher overall rate of cancers in anti-RNA Pol III positive patients compared to controls (anti-RNA Pol III negative) (17.7% vs 9.0%, *p* = 0.015) with a higher incidence of neoplasms synchronous with SSc (OR: 7.38%). Regarding cancer types, the incidence of solid neoplasm, in particular breast malignancy, was higher in anti-RNA Pol III positive patients. Furthermore, authors reported a greater proportion of men among patients diagnosed with non-breast synchronous cancer [68]. More recently, Lertphanichkul and Smith [57] confirmed the significant higher incidence of cancers in patients with anti-RNA Pol III antibodies compared to anti-topo I and ACA positive patients. In the study of Morrisroe et al. [19] the association between anti-RNA Pol III antibodies and cancer was again demonstrated and, when diagnosed within 5 years of SSc onset, cancer was more likely to occur in older and anti-RNA Pol III positive patients, particularly regarding breast cancer [19]. In the case–control study on 2.431 SSc patients and 12.710 control subjects, Watad et al. [69] showed an increased risk of cancer in patients with anti-topo I and anti-RNA Pol III abs [69]. In addition, the study suggested that humoral autoimmunity represented by autoantibodies status may impact survival in SSc patients with cancer. Particularly, antinuclear antibodies (ANA) negativity, anti-topo I and anti-RNP positivity appeared to be associated with a less favourable outcome, although the hazard ratio for death was significant only for ANA and anti-topo I antibodies. In addition, among SSc patients positive for anti-topo I, those with cancer were reported to have an increased risk of death compared to those without [69]. The significant higher prevalence of cancer in anti-RNA Pol III positive subjects has not been confirmed by all studies [70–72]. Comparing SSc patients with anti-RNA Pol III positivity and those without, a recent study did not observe a significant different prevalence in cancer occurrence between the two groups of subjects [72]. All together data about anti-RNA Pol III antibodies reported a higher incidence of cancer in patients with these antibodies with a close temporal relationship between malignancy occurrence and SSc onset. These findings lead to obvious implications in clinical practice, suggesting regular screening and follow-up for patients with anti-RNA Pol III antibodies [68]. In addition, the close temporal relationship between SSc and cancer may lead to consider SSc as a paraneoplastic manifestation. In this context, Joseph et al. [73] found that out of 16 SSc patients with cancer, 8 were positive for anti-RNA Pol III antibodies. In 5/8 cases, cancer occurred before SSc onset and in the remaining 3 patients it developed within the 2.5 years of SSc onset. Authors confirmed a major temporal gap between SSc beginning and cancer in patients with anti-topo I and ACA positivity. Authors analysed neoplastic tissue and reported somatic genetic alterations of the POLR3A locus, encoding polymerase III, in 6/8 patients with anti-RNA Pol III antibodies and none in TOP1 or CENPB. Alterations in POLR3A were not found in patients without anti-RNA Pol III antibodies. According to these findings, the gene mutations located in tumor cells could trigger an immune response with the exposure of new self-antigens leading to the production of autoantibodies that started a humoral and cellular response typical of autoimmune rheumatic disease as SSc [73].

Concerning the risk of cancer, the role of SSc-specific antibodies other than anti-RNA Pol III still remains uncertain and debated [69, 74]. As above reported, also anti-topo I antibodies positivity seems to be associated with a higher frequency of cancer among SSc patients [34] and these antibodies have been suggested to be a hallmark for the occurrence of malignancy in SSc [33, 75]. Already in 1996, Kuwana et al. reported an increased levels of anti-topo I antibodies in two SSc patients after the diagnosis of lung adenocarcinoma. In addition, sera obtained from patients after cancer diagnosis, recognized some novel and/or different epitopes of the entire topoisomerase I molecule suggesting that autoantibodies specificities may change after malignancy occurrence [76].

As previously mentioned, another autoantibody subset that has been associated with cancer occurrence is the “triple negativity” [67, 77, 78]. In patients without neither anti-RNA Pol III nor anti-topo I nor ACA, a certain increased frequency of cancer occurrence has been reported. As already described, Igusa et al. [62] reported an increased risk of melanoma and breast cancer among these patients and older age has been demonstrated to represent a risk factor for malignancy development particularly in these SSc subjects [62, 67]. In contrast with these results, findings from a recent study suggested a protective role of SSc-specific antibodies negativity on the occurrence of haematological cancers by the multivariable logistic regression analysis [19]. However, most studies seem to be in accordance suggesting an association of triple negativity and cancer and this datum does not refute the suspected relationship between autoimmunity and cancer, rather it might bring to suspect the presence of another unknown autoantibodies subset predisposing cancer occurrence [79]. 

Among SSc-antibodies identifying patient subgroups at major risk to develop cancer, also anti-PM/Scl antibodies seem to be associated with an increased occurrence of neoplasia. Bernal-Bello et al. [71] showed presence of cancer in 53/432 SSc enrolled patients describing a more frequency of anti-PM/Scl in patients with cancer and a significant increased risk of neoplasm in patients with these antibodies (OR = 3.9; 95% CI 1.31–11.61; *p* = 0.014) [71]. However, this datum has not been confirmed by all studies, and for example the analysis on 46/305 SSc patients with cancers showed a similar prevalence of anti-PM/Scl antibodies in SSc patients with and without neoplasm (6.5% vs 6.9%, *p* = 0.916) [80].

Data regarding the relationship of ACA and anti-Th/To antibodies suggested a decreased frequency of cancers in patients with these autoantibodies, compared with general population. ACA positivity is generally reported to be associated with a lower frequency of cancer occurrence and also in oncological patients without rheumatic diseases, the presence of ACA positivity is shown to correlate with a good prognosis. In this context, Atalay et al. [81] evaluated a population of 55 patients with breast cancer and without diagnosis of connective tissue disease and suggested anti-centromere protein (CENP)-B antibodies positivity as a prognostic factor for disease-free survival and overall survival [81]. Morrisroe et al. suggested a protective role of ACA positivity in lung cancer occurrence with an OR of 0.22 (*p* = 0.023) [19].

A protective role of anti-Th/To for cancer development among SSc patients has also been suggested. In the recent study of Mecoli et al. [82] a negative association between the positivity for each Th/To antibody specificity (hPOP1, RPP25, RPP30, and RPP40) and cancer was demonstrated. In addition, authors also suggested a possible protective effect of anti-Th/To antibodies that seemed able to modify cancer risk given by anti-RNA Pol III positivity; however, this finding requires additional analysis on larger populations as in this study only 9 patients presented both anti-Th/To and anti-RNA Pol III antibodies [82].

Table 2 summarizes the most relevant data on cancer risk associated to SSc-specific autoantibodies.

**Table 2:** Association between autoantibodies in systemic sclerosis and cancer risk

Autoantibody	Incidence of cancer (%)	Absolute SSc-cancer interval (year)	Type of cancer associated
Anti-RNA Pol III	14.2-20.9	5.3 (2.6-10.8)	Breast cancer Lung cancer
Anti-topoisomerase I	6.3-13.6	8.4 (1.8- 16.5)	Lung cancer Haematological neoplasms
Anti-centromere antibodies (ACA)	6.8-16	9.2 (3.7-17.9)	Gastrointestinal cancers, thyroid cancer Protective role for lung cancer
Triple negative	7.7-14.9	10.8 (4.3- 16.7)	Breast cancer Melanoma
Anti-PM/Scl	~9.3	14.0 (13.2- 34.6)	Undefined relationship with cancer
Anti-Th/To	6.9	27.0 (22.0- 32.0)	Protective role for cancer development

Anti-RNA Pol III: anti-RNA polymerase III; Triple negative: negative for ACA, anti-topo-I and anti-RNA Pol III

### Immunosuppressive Treatment in SSc Patients and Cancer Risk

Several immunosuppressive therapies have been approved for the treatment of patients with SSc [83] and such agents might play a role in the development of malignancy, although data are contradictory and often derived from studies on other rheumatic and non-rheumatic diseases.

Cyclophosphamide, an alkylating agent, is the cornerstone of the treatment of SSc, in particular of ILD and skin involvement related to SSc [84, 85]. As above mentioned, long-term treatment with cyclophosphamide is associated with an increased risk of developing transitional cell bladder carcinoma [56] and haematological malignancies [86]. The risk of developing malignancy is dose-dependent, and the benefit from cyclophosphamide treatment must be weighed against the risk of long-term AEs [87].

Mycophenolate mofetil (MMF) has been shown to have comparable efficacy to cyclophosphamide with a better toxicity profile [85, 88] particularly in treatment of SSc lung involvement. Cancer risk data related to MMF are mostly derived from transplant patients, with contradictory results on the increased risk of lymphoproliferative and non-melanoma skin cancer [89–92]. However, no conclusions can be drawn on the risk of cancer-related to the use of MMF, because many of the transplant patients receive multiple immunosuppressive treatments.

Methotrexate is mainly used for forms with widespread skin involvement and in patients with arthritis [84] and it is associated with an increased risk of non-melanoma skin cancer, although these data come from a study on patients with RA or psoriasis [93].

The use of azathioprine appears to be associated with an increased risk of non-melanoma skin cancer [94, 95], but with conflicting results: a meta-analysis of 5 studies of patients receiving long-term treatment for myasthenia gravis did not show an increased risk of neoplasms [96]. However, no data are available on the risk of cancer for patients with SSc treated with aziathioprine.

A recent study on an Italian cohort of SSc patients treated with immunosuppressants confirmed an increased incidence of neoplasms in SSc patients without finding an association between cancer risk and exposure to immunosuppressive drugs commonly used for the treatment of SSc [97].

Data on the new biologic disease-modifying drugs are contradictory and derive from studies on their use in other rheumatic diseases. Some studies have shown an increased risk of invasive melanoma [98, 99], and an increased risk of squamous cells skin cancer in patients treated with abatacept [100].

Tocilizumab, a humanized anti-interleukin-6 receptor antibody that might preserve lung function in patients with early SSc-ILD [101], does not seem to increase cancer risk. In a large Swedish cohort study of rheumatoid arthritis (RA) patients, the overall cancer risk was not increased by tocilizumab [100]. Furthermore, data from a large European collaborative project do not show an increased risk of invasive melanoma in patients treated with tocilizumab for RA [99]. Longer follow-up data on tocilizumab use for SSc-ILD could clarify whether there is an increased risk of cancer.

Further prospective studies in SSc patients are needed to define cancer risk related to these drugs; in the meantime, the choice of the best treatment must be guided by the balance of risks and benefits, and adequate monitoring of patients is recommended in order to diagnose neoplasms early.

On the other hand, drugs used for the treatment of SSc can also have an antineoplastic effect. This is the case of nintedanib, a tyrosine kinase inhibitor, that when used in the SSc-ILD reduces the rate of decline in forced vital capacity [102]. In combination with docetaxel, nintedanib demonstrated an advantage in progression-free survival and overall survival in patients with lung adenocarcinoma, when used as second-line therapy [103].

## Common Mechanism in SSc and Cancer

### Genetic and Epigenetic

#### Telomere Shortening

The impact of telomere attrition is well established in cancer while in autoimmune diseases remains still unclear although autoantibodies against many telomere nucleoprotein components are prevalent in the latter. It has been noted in SSc telomere shortening [104, 105] and the study by Fujji et al. [106] has hypothesized that telomerase insufficiency in rheumatoid arthritis (RA) patients results in excessive T-cell loss, impairing homeostatic control of the naive T-cell compartment. Thus, telomerase insufficiency if confirmed in prospective studies could represent a therapeutic target for resetting immune abnormalities in patients affected by autoimmune disorders. In patients with SSc with lung disease, autoantibodies targeting telomere-associated proteins have been identified and are associated with short telomeres in circulating lymphocytes [107].

#### miRNA

Emerging evidence suggests a possible role of miRNAs in the pathogenesis of SSc; however, to date contradictory results have been reported. Recently a meta-analysis revealed a small cluster of differentially expressed miRNAs, of which miR-21 in blood, miR-29a, miR-155, and miR-196a in dermal fibroblasts, and let-7a in both serum and dermal fibroblast samples [108]. miR-21 exert key roles in the pathogenesis of fibrosis and cancer: in SSc miR-21 enhances TGF-β signalling inducing fibrosis [109] and additionally miR-21 is one of the first oncomiRs found upregulated in several cancers and represents a plausible diagnostic and prognostic biomarker, as well as a therapeutic target [110]. A prospective pilot case–control trial is ongoing (NCT04148716) that evaluates miRNA profiles in SSc tissues and in particular is evaluating pro-fibrotic “key” miRNAs called FibromiRs (miR-199a-3p, miR-199a-5p, and miR-214) associated with monitoring the response to TGF-β in fibroblasts. miR-214-3p is downregulated in lung cancer patients and acts as a vital target in FGFR1-amplified patients by forming a miR-214-3p-FGFR1-Wnt/MAPK/AKT signalling pathway network. More importantly, miR-214-3p is correlated to a favourable patient prognosis and acts as a biomarker to predict chemotherapy response and outcome [111]. Another miRNA strongly involved both in cancer and fibrosis is let-7d that is as a key regulator of cell proliferation and can act as a tumor suppressor [112]. It is also involved in the regulation of EMT and prevention of lung fibrosis [113]. The expression of let-7d is downregulated in SSc skin [114]. Furthermore, additional cancer-related miRNAs (breast, lung, and haematological malignancies) are deregulated in SSc patients: expression levels of miR---21­--5p, miR­---92a­--3p, miR-155--­5p, and miR-­16­--5p are higher in SSc sera compared to healthy controls [115].

#### LncRNA

Though it was largely recognized that lncRNAs exert a key role in the regulation of autoimmune diseases [116], few data on lncRNAs in SSc are available. Dolcino et al. [117] have characterized the expression profiles of lncRNAs in SSc patients and find out that a unique lncRNA, namely heterogeneous nuclear ribonucleoprotein U processed transcript (ncRNA00201), is deregulated in SSc. Its gene target Heterogeneous nuclear ribonucleoproteins C (hnRNPC) encodes for a known autoantigen in SSc [118]. It is noteworthy that ncRNA00201 has been reported to be involved in cancer proliferation [119] reinforcing the hypothesis of a link between SSc and tumor development.

### Signalling Pathways

#### Glycolysis

The role played by glycolysis in the differentiation of fibroblasts and in fibrotic diseases is rapidly emerging. In the lung tissue of patients with idiopathic pulmonary fibrosis (IPF), disruption of amino acid metabolism and glycolysis has been evidenced and glycolytic enzymes (including PFKFB3, PFK1, and HK2) are upregulated [120, 121]. Furthermore, recently a pathway enrichment analysis showed an enrichment in signalling network of glycolysis in SSc samples [115] in a similar way to what has been extensively described in cancer patients [122].

#### Oxidative Stress

Cancer cells exploit aberrant redox homeostasis and are influenced by reactive oxygen species (ROS) in a contradictory way: low ROS levels support transformation/proliferation of cancer cells, and high ROS levels are cytotoxic [123]. The importance of the role of oxidative stress in the aetiology of SSc was demonstrated by Murrel in 1993 [124] and over the last three decades became clear that oxidative stress plays a key role in its pathogenesis. ROS act on different cellular targets of SSc, such as activation of endothelial cells, differentiation/proliferation of fibroblasts, and on the fibrosis activating the synthesis of ECM proteins [125]. Moreover, advanced oxidation protein products (AOPPs), which provide indirect evidence of oxidative stress, are upregulated in SSc and induce endothelial cells and fibroblasts to produce hydrogen peroxide (H2O2) and are involved in vascular and fibrotic complications [126]. Thus, oxidative stress and antioxidant molecule balance is critical for fibroblast activation and function also in SSc.

#### PI3K/Akt

The PI3K/Akt pathway not only exerts a relevant role in lung fibrosis as emerged from preclinical in vitro and in vivo models [127, 128] but also is involved in cancer development [115, 129]*.*

### Other

#### Microbiota

The correlations between microbiota dysbiosis and cancer have gained extensive attention and been widely explored and recently several studies have demonstrated variable degrees of dysbiosis in numerous autoimmune diseases [130]. SSc disease state has been associated with alterations in the gastrointestinal tract microbial consortium: specific faecal microbial taxa were altered (enriched or depleted) in patients with SSc compared with healthy controls. Regarding phylum-level differences, the relative abundance of Bacteroidetes was decreased in the SSc patients [131]. Recently an extensive characterization of gut microbiome ecology in SSc has been reported [132].

## Immune Balance: Autoimmune Disease and Cancer

The delicate balance of the immune system that normally prevents damage to the self is regulated by co-stimulating and co-inhibiting molecules, also known as immune checkpoints. Over the past decade, activation of the immune system by inhibition of downregulating-immune checkpoints has been at the centre of new developments in cancer treatment. Indeed, checkpoint-inhibiting therapies, eliminating inhibitory blockade, and promoting activation of cytotoxic T cells can counteract cancer growth. The knowledges derived from the use of immune checkpoint inhibitors (ICIs) in cancer therapy allowed the discovery of an increasing number of new immune checkpoint receptors and ligands, providing an interesting approach to study their implication in the pathogenesis of autoimmune diseases and their potential therapeutic role.

### Checkpoint-Blocking Therapy in Cancer and Adverse Effects of Checkpoint Inhibitors

The immune system plays a role in both the development and treatment of cancer. The most pivotal shift in use of the immune system in the fight against cancer came with the discovery of ICIs. Malignant tumors take advantage of the inhibitory programmed cell PD-1/PD-L1 or CTLA-4 pathways to evade the immune system [20]. The development of drugs promoting the disruption of this axis by blocking monoclonal antibodies allowed durable remissions in different types of cancer that previously were considered incurable. Indeed, substantial improvements in terms of survival have been documented in patients with metastatic cancer suggesting the ground‑breaking impact of immune modulation across different tumors [133–135].

Since 2011 the Food and Drugs Administration (FDA) approved an antibody against CTLA-4 (ipilimumab), two antibodies against PD-1 (pembrolizumab and nivolumab), and against PD-1 ligand 1 (atezolizumab and durvalumab) for cancer treatment. The combination of anti-PD-1 and anti-CTLA-4‑agents further improves clinical response rates compared with single‑agent activities in some types of cancers [136]. The superiority of combination therapy is most likely due to the different and non-redundant mechanism by which CTLA-4 and PD-1 inhibit T cells. In fact, CTLA-4 is expressed by activated T cells and competes with CD28 for costimulatory ligands attenuating the early activation of naive and memory T cells [137]. The use of CTLA-4 inhibitors causes an increase in T cell infiltration into tumors and reduces it at the level of the tumor microenvironment, preventing the suppression of cytotoxic T cell activity [138]. PD-1 directly interferes with the T cell receptor signalling at the effector stage within tissues by inhibiting and causing depletion [139]. Tumors expressing PD-1 ligands thus protect themselves from T cell–mediated killing [140]. However, additional checkpoint‑blocking approaches such as V‑type immunoglobulin domain‑containing suppressor of T cell activation (VISTA) or T cell immunoreceptor with Ig and ITIM domains (TIGIT) blocking for the treatment of human cancer are expected to demonstrate efficacy in some kind of solid tumors.

Although ICIs have a beneficial role in the activation of T cells directed against the tumor antigen, they can also lead to aberrant activation of T cells reactive to autoantigens, resulting in side effects that resemble autoimmune diseases. In fact, both CTLA-4 and PD-1 block are associated with side effects known as immune-related adverse events (irAEs), whose underlying mechanisms still remain unclear. These irAEs can affect any organ, but typically the skin, intestines, liver, and endocrine organs, as in autoimmune diseases [141]. Although some irAEs have been well‐documented, there is a little knowledge of rheumatic irAEs, including arthralgia, arthritis, myositis, polymyalgia–rheumatica‐like (PMR‐like) syndrome, sicca syndrome, vasculitis, and scleroderma. In general, the incidence and severity of irAEs are more marked among patients treated with anti-CTLA-4 or combination of anti-CTLA-4 and anti-PD-1, than in those treated with anti-PD-1 or anti-PDL-1 alone [141]. This could be attributed to the difference in the T cell activation process. In fact, the use of anti-CTLA-4 unblocks T cells at an earlier stage of their development compared to PD-1 inhibitors with a consequent increase in the frequency of autoreactive T cells. This difference could also explain the further increase in the severity of the irAEs observed among patients treated with combination therapies [141, 142]. The most frequent adverse manifestations within the first few weeks of treatment include rash and/or pruritus on the patient’s trunk and extremities [143]. Gastrointestinal irAEs are common and usually first occur 4–6 weeks after initiation of treatment [141]; hepatic irAEs are observed less frequently and occur in approximately 5% of patients treated with CTLA-4 and PD-L1 block. The most common endocrine disorders include CTLA-4 blocking hypophysitis and anti-PD-1-induced hypothyroidism, affecting approximately 10% of patients. Although most irAEs are reported as moderate and relatively simple to treat with temporary interruption of the therapy or mild immunosuppressive treatment as systemic corticosteroids, in some cases, irAEs are fatal, as reported in organizing inflammatory pneumonia and myasthenia gravis [144, 145]. Since ICIs treatment can induce a lasting response in metastatic disease, unlike traditional chemotherapy, it is important to be aware that in patients experiencing serious side effects, the discontinuation of treatment can lead to a reduction of efficacy and to a treatment failure. Because of this, availability of effective therapies against irAEs is expected, and this concept emphasizes the fact of the strong connection between cancer immunotherapy and autoimmune disease.

### ICI in Pre-existing Autoimmune Diseases (PAD)

As stated above, ICIs are associated with a broad spectrum of immune AEs which appear more pronounced in patients with pre-existing autoimmune diseases (PADs). In this context, the use of ipilimumab is reported to cause exacerbation in approximately one quarter of patients with pre-existing autoimmune disorders [146]. In these patients, ICI activation of the immune system may result in more severe irAEs due to their underlying abnormal immune response to self-antigens. For this reason, patients with PAD were excluded from large randomized controlled trials evaluating the efficacy and safety of ICIs and only limited data from case series/case reports are available [147–149]. Also patients with only specific autoimmune autoantibodies are reported to be more likely to develop irAE if treated with anti-PD-1 antibody [147, 148, 150].

A systematic review of 123 pre-existing cancer and autoimmune diseases patients treated with ICI reported exacerbation of PAD, development of irAE, or both events in 75% of patients. Of these, 41% had recurrence or worsening of previous manifestations, 25% developed *de novo* irAE, and 9% had both. Patients with PAD on active treatment at the time of initiation of ICI therapy had fewer AEs than those who were not receiving treatment (59% vs 83%) [151]. In a prospective study by Danlos *et al*., irAEs developed more frequently and more rapidly in patients with PAD than in patients without PAD (44% versus 24%) [152]. Similarly, in a retrospective study by Cortellini *et al*., the incidence of any grade irAEs was higher among patients with PAD than in patients without PAD (66% vs 40%), with no difference in the incidence of grade 3–4 irAE among these groups (9.4% vs 8.8%) [153]. Another retrospective study of patients with PAD treated with ICIs reported the irAEs development in 38% of patients who required glucocorticoids and discontinuation of ICIs. 63% had previously received a disease-modifying anti-rheumatic drugs **(**DMARDs), but only 2 patients were on active systemic treatment at the time of ICI initiation. A longer survival was observed in patients who experienced irAEs than those who did not have an irAE [154]. Leonardi *et al.* conducted a retrospective study in patients with PAD (with active symptoms and on immunosuppressant or immunomodulatory agents) treated with a PD-1/PD-L1 inhibitor. Grade 1-2 exacerbations of underlying PAD occurred in 23% of patients and were more frequent in patients with rheumatologic disorders compared with other PAD. Treatment included supportive care and steroids. Partial responses (PR) and stable disease (SD) were recorded in 22% and 31%, respectively [155].

Recently, 27 patients with PAD and cancer who reported exacerbations of disease during antiPD-1 immunotherapy were evaluated in a national case series from the Canadian Research Group of Rheumatology in Immune-Oncology (CanRIO) [156]. Nearly 80% of patients developed at least one irAE, usually mild and manageable, but required ICI to be discontinued in one third of cases. The most observed PADs were RA, psoriasis/psoriatic arthritis, immune bowel disease, and axial spondyloarthritis while, for tumors, lung cancer and melanoma. Exacerbations were more frequent and/or severe in patients requiring more intensive pre-ICI systemic therapy and occurred despite the preventive use of immunosuppressive drugs prior to ICI treatment. As for the efficacy outcome, more than 40% of patients with PAD vs 15% without PAD presented tumor progression at a median follow-up of 11 and 17 months, respectively [156]. The irAEs were more frequent in patients without tumor progression confirming the positive predictive role of irAE on the antitumor response as already previously reported [157]. Tumor progression was not associated with exposure to immunosuppressive drugs before or after the onset of ICI. Furthermore, the rate of severe irAEs leading to ICI discontinuation was lower in patients with PAD (33% vs 52%) thus not explaining the higher rate of progression observed in these patients [158]. Contrarily, another study observed a shorter median progression-free survival among patients with PAD who received immunosuppression at the onset of ICI in a multivariate analysis adjusted for gender, age, cancer type, and ICI type [159]. Likewise, Menzies et al. [160] reported, in an analysis adjusted for cancer stage, brain metastases, performance status, and/or LDH, lower response rates among immunosuppressed patients at the onset of ICI (15% vs. 44%). Steroid use at prednisone-equivalent doses of ≥ 10 mg at the time of ICI initiation has also been associated with poor cancer outcomes [161, 162]. Thus, although data on ICI therapy in patients with PAD and cancer are still debated, guidelines from the National Comprehensive Cancer Network (NCCN) recommend considering ICI in patients with low or no immunosuppression and a good control of PAD, avoiding them, if possible, in life threatening or poorly controlled PAD or in patients requiring high levels of immunosuppression [163].

### Targeting Checkpoints in Autoimmunity

**Fig. 1:**
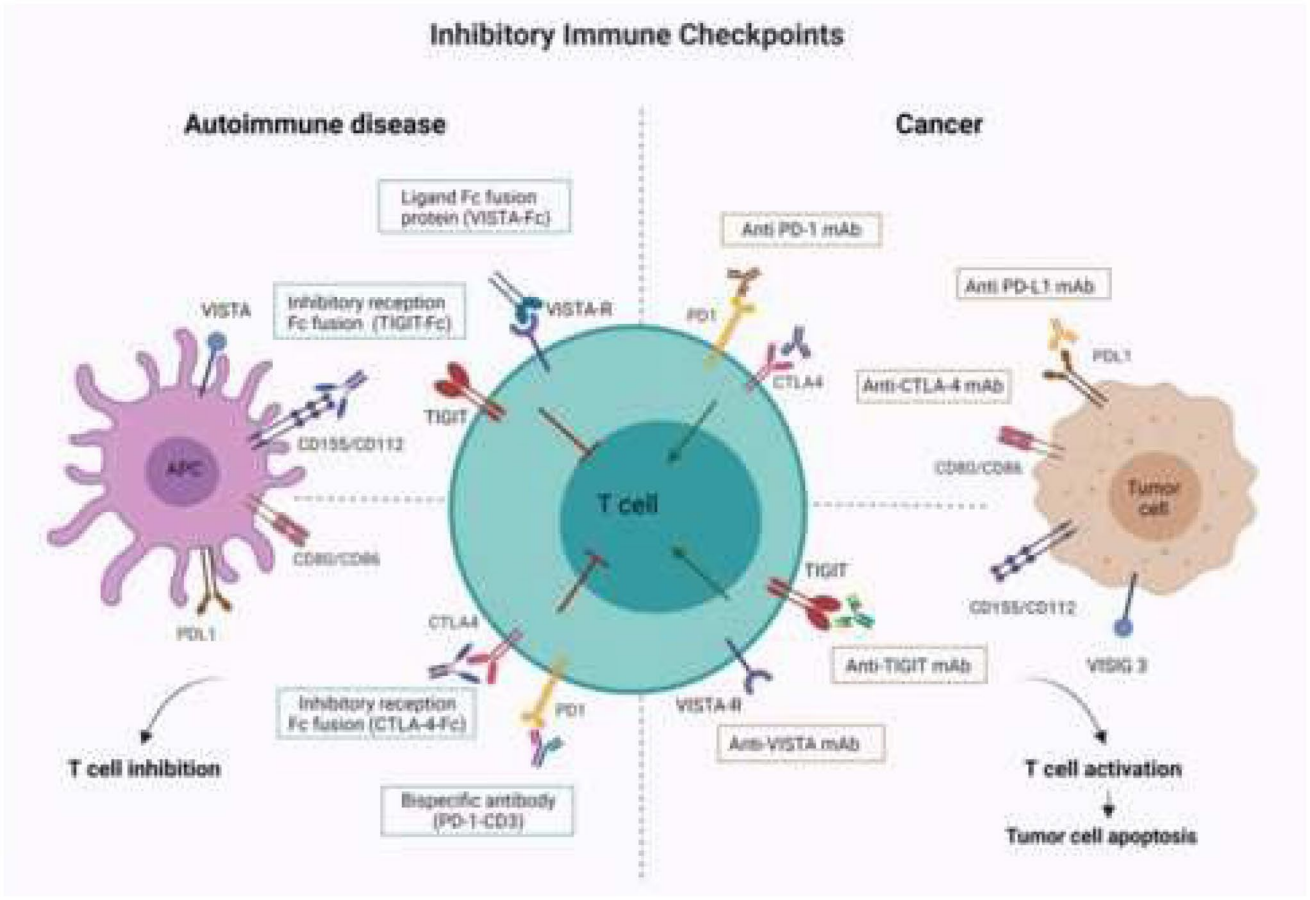
Inhibitory immune checkpoints targeting. Multiple approaches are available to target immune checkpoints for the treatment of autoimmune disease or cancer. Shown on the right example of immune checkpoints inhibitors (ICIs) approved for the treatment of cancer (e.g., antibodies against PD-1, PD-L1 and CTLA-4) and additional emergent checkpoint-blocking approaches (e.g., antibodies against TIGIT and VISTA). These antibodies negatively regulate the inhibitory immune receptors keeping the immune response active and promoting kill of tumor cells. On the left some specific examples of inhibitory receptors that have been targeted in preclinical and clinical studies: soluble inhibitory-receptor-Fc fusion protein (CTLA-4-Fc and TIGIT-Fc ) ligand-Fc fusion protein (VISTA-Fc), bispecific antibodies (PDI-CD3). Contrary to the principle of cancer immune theraphy with ICIs, these methods keep the immune response off improving the course of the autoimmune diseases. mAB, monoclonal antibody; PD-1, programmed dealth protein 1; PD-L1, PD-1 ligand; CTLA-4, cytotoxic T-lymphocyte antigen 4; TGIT, T cell immunureceptor with Ig and ITIM domains; VISTA, V-type immuniglobulin domain-containing suppressor of T-cell activation, VISTA-R, VISTA receptor, Image created with BioRender.com

The experience of cancer treatment with ICIs and the study of related irAEs have provided an interesting opportunity to study the early biology of autoimmune diseases and to design new treatment options for these conditions. Considering the principle on which immune therapy in cancer is based and the role that ICIs play in autoimmune rheumatic diseases, all inhibitory receptors could represent potential targets for therapeutic interventions in autoimmune diseases. To date, multiple approaches are available for targeting immune checkpoints for the treatment of autoimmune rheumatic diseases, including soluble inhibitory receptor-Fc fusion proteins, ligand-Fc fusion proteins, artificial ligands, agonistic antibodies, and bispecific antibodies, which bind an inhibitory receptor and activator (Fig. 1). Abatacept, a CTLA-4-Fc fusion protein, is the first checkpoint targeted drug approved for the treatment of rheumatic diseases. CTLA-4-Fc binds in the same way as CTLA-4 to the high affinity costimulatory ligands CD80 and CD86 preventing their costimulatory signalling [164]. It is currently used in patients with RA and juvenile idiopathic arthritis (JIA), although CTLA-4-Fc clinical efficacy is being tested on other autoimmune rheumatic diseases such as SSc [165]. Several other inhibitory receptors that modulate T cell activation have been explored as potential therapeutic targets in autoimmune diseases. Among these, TIGIT which is expressed by T cells and natural killer cells and binds CD155 on dendritic cells (DCs), macrophages with high affinity and CD112 with lower affinity [166, 167]. The binding with its ligand on DC results in an increase in the secretion of IL10 by DCs and a decrease in the proliferation of T cells. The blockade of TIGIT determines a powerful antitumor immune action [168–170]. Co-blockade of TIGIT and PD-1 pathways elicits tumor rejection in preclinical murine models. TIGIT is also involved in autoimmune diseases and appears to inhibit pro-inflammatory immune responses that drive organ-specific autoimmunity as demonstrated in a preclinical study where administration of soluble TIGIT or anti-TIGIT agonist antibodies in mice with collagen-induced arthritis reduced the severity of the disease [171]. The PD-1 pathway appears to be negatively linked with the development of several autoimmune diseases [172–174] and several targeted treatment approaches have been studied in mouse models [175]. Depletion of PD-1 positive cells appears to lead to the improvement of autoimmune diseases including type I diabetes and experimental autoimmune encephalomyelitis [176]. Therefore, targeting PD-1 could be a promising strategy for treating these diseases. VISTA could represent another potential target for T cell inhibition in autoimmune rheumatic diseases. It binds non-better characterized inhibitory receptor expressed on T cells causing its inhibition. The presence of VISTA-Fc patterns in vitro resulted in inhibition of T cell proliferation and cytokine production, while blocking VISTA and failure to bind to T cells improved T cell responses [177]. Therefore, targeting different inhibitory receptors alternately or simultaneously could aid the successful treatment of patients with autoimmune rheumatic diseases, even in those unresponsive to certain targeted checkpoint therapies. Finally, an alternative approach to fight autoimmune rheumatic diseases could be the targeting of inhibitory receptors expressed on immune cells other than T cells belonging to both the innate and the adaptive arm of the immune system. An interesting candidate seems to be FcγRIIB, an inhibitory receptor linked to rheumatic diseases whose block by bispecific antibodies has shown improvement in autoimmune diseases in preclinical studies [178].

In conclusion, although the available evidence for the use of ICI in patients with pre-existing autoimmune diseases is limited and derived from retrospective analysis, for the majority of the PAD, the use of ICIs appears to be safe and effective. Therefore, the treatment with ICI should be considered for patients with severe PAD including those with an active disease. However, special consideration should be given to patients receiving high levels of immunosuppression for PAD control because the immunosuppression might compromise efficacy of ICIs. Moreover, multidisciplinary approach with the involvement of an autoimmune disease’s specialist and a good assessment of the PAD activity and organ impairment before the starting of an ICI should be recommended. Moreover, a multidisciplinary approach to every irAE in patients with PAD could facilitate the management of potential fatal irAEs [179–181]. In fact, the exacerbation of the underlying condition if not adequately controlled can lead to a potential life-threatening event or to the definitive interruption of treatment with a strong impact on survival.

## Cancer Surveillance in SSc Patients

The strong association between SSc and cancer led to suggesting surveillance and periodical malignancy screening in SSc patients. As previously discussed, some serological and clinical features, as older age and anti-RNA Pol III antibodies positivity, may identify subjects at risk to develop cancer synchronously with SSc onset. These data could be of primary importance, also suggesting when to perform and to repeat neoplastic screening in patients with risk factors. Shah and Casciola-Rosen [182] proposed a screening algorithm according to the presence of specific risk factors, recommending to perform all neoplastic screenings already indicated in age- and gender-matched general population also in SSc patients and indicating annual mammography from 40 years of age. Regarding the increased frequency of breast cancer in SSc, other studies have suggested to subject all anti-RNA Pol III female patients with a diffuse SSc subset to breast magnetic resonance (MR) and to closely monitor patients with these clinical and autoantibodies profile for tongue, lung, and prostate cancers [62]. In addition, chest, abdomen, and pelvis computed tomography (CT) or positron emission tomography (PET) may be indicated in older patients at SSc diagnosis, subjects with anti-RNA Pol III antibodies, when the disease is unresponsive to common treatment, if patients complain important constitutional symptoms like fever or weight loss and also in subjects with cancer history in family members [182]. A tight surveillance of patients treated with alkylating agent as well as of subjects with cytopenia or monoclonal gammopathies or with precancerous lesions, as Barrett’s oesophagus, or liver cirrhosis (for instance in SSc patients with associated primary biliary cholangitis) is also recommended [183]. Patients with persistent and unresponsive to treatment gastroesophageal reflux or with unexplained dysphagia should be investigated with upper endoscopy and otolaryngology evaluation, in order to early detect cancerous or precancerous lesion of the oesophagus, pharynx, or togue that have been reported among SSc patients [61, 183]. Although a more aggressive algorithm of cancer screening in some SSc autoantibody subsets (anti-RNA Pol III and triple negative patients) seems to be suggested, the risk–benefit ratio of this tight screening surveillance, particularly regarding radiation exposure with CT and PET use, has yet to be elucidated [40, 79]. Through a Delphy exercise involving eighty-two experts in the third stage, a study on EUSTAR database proposed a possible cancer screening algorithm for SSc patients with anti-RNA Pol III antibodies [68]. With a high agreement level, authors suggested to perform mammography in women (and/or MR/ultrasound), to screen for the possible presence of other neoplasms with non-invasive tests that should be considered in all patients (faecal occult blood, gynaecological evaluation, prostatic-specific antigen, ultrasound evaluations). In addition, to monitor patients in the first year from SSc onset was also recommended with a high level of agreement; in addition, experts suggested a tight surveillance for cancer development for a subsequent period of 2–5 years. Particularly, in case of negativity of all tests at the beginning of SSc, breast examination was proposed to be annually repeated. The other screening tests should be performed again in case of presence of suspected signs or symptoms. However, also in this study authors highlighted the need of further prospective studies to clarify if and when more invasive examinations, such as CT and PET, should be used and how to survey patients during a longer follow-up [68]. Altogether these data indicate to perform a cancer screening in all SSc patients and particularly patient’s age and autoantibody subset should drive clinicians in patient management and surveillance. According with the most shared screening algorithm, clinical and instrumental examinations indicated for age- and sex-matched general population have to be performed at baseline, regularly repeated and repeated in case of signs or symptoms suspected for neoplasia in all SSc patients. In addition, breast and prostate evaluations and ultrasound examinations have to be considered at SSc diagnosis together with all the other non-invasive tests. Only patients with risk factors or signs/symptoms suspected of malignancy should be investigated with CT or PET, after an appropriate evaluation of risk and benefit. In addition, when performing paraneoplastic screening, the described biphasic relationship between cancer and SSc described at the beginning also has to be considered.

## Conclusions

Altogether the reported data highlight the tight association between SSc and cancer. The identification of common pathogenetic mechanisms triggering the two diseases, and the detection of risk factors both for cancer occurrence in SSc and for rheumatic autoimmune disease development in cancer patients should strengthen an active collaboration between the rheumatologist and the oncologist. The importance of an early diagnosis is now well known both in SSc and in cancer, and in this context a joint and shared management between rheumatologist and oncologist becomes mandatory to treat early patients in their window of opportunity trying to achieve the best result in terms of survival.
